# Analysis of risk factors and predictive efficacy of senile osteoporosis fracture based on biochemical indicators of bone metabolism

**DOI:** 10.5937/jomb0-46663

**Published:** 2024-06-15

**Authors:** Yufang Mao, Kanghua Li, Bing Zhu, Jiang Long

**Affiliations:** 1 Zhuzhou Peopležs Hospital, Sports Medicine, Zhuzhou, China; 2 Zhuzhou Peopležs Hospital, Department of Rehabilitation Medicine, Zhuzhou, China; 3 Zhuzhou Peopležs Hospital, Department of Orthopaedics, Zhuzhou, China

**Keywords:** bone metabolism, osteoporotic fracture in the elderly, risk prediction, predictive efficacy, metabolizam kostiju, osteoporotski prelom kod starijih osoba, predviđanje rizika, prediktivna efikasnost

## Abstract

A total of 254 elderly OS patients diagnosed and treated in our hospital during May 2019 to April 2022 was randomly picked, of which 100 patients were finally enrolled. Patients were divided into OS fracture group and non-fracture group according to whether they had OS fracture. The contents of bone mineral density (BMD) and bone metabolism biochemical indexes, including Dickkopf1 (DKK-1), sclerostin (SOST), osteoprotegerin (OPG), osteopontin (OPN), osteocalcin (BGP) and 25 hydroxyvitamin D (25 (OH) D) were detected in lumbar L2č4 and left femoral greater trochanter. The correlation between bone metabolism and BMD was evaluated using Pearson analysis. The risk factors of OS fracture were analyzed using Multivariate logistic regression analysis. The predictive value of biochemical indexes of bone metabolism on the risk of OS fracture was analyzed using ROC curve.

## Abstract (sr)

**Metode:**

Nasumično je izabrano ukupno 254 starijih pacijenata sa OS dijagnostikovanim i lečenim u našoj bolnici u periodu od maja 2019. do aprila 2022. godine, od kojih je 100 pacijenata konačno upisano. Pacijenti su podeljeni u grupu sa prelomom OS i grupu bez preloma prema tome da li su imali frakturu OS. Sadržaj mineralne gustine kostiju (BMD) i biohemijskih indeksa metabolizma kostiju, uključujući Dickkopf-1 (DKK-1), sklerostin (SOST), osteoprotegerin (OPG), osteopontin (OPN), osteokalcin (BGP) i 25 hidroksivitamin D (25 (OH) D) otkriveni su u lumbalnom L2č4 i levom femoralnom velikom trohanteru. Korelacija između metabolizma kostiju i BMD je procenjena korišćenjem Pearsonove analize. Faktori rizika preloma OS analizirani su primenom Multivarijantne logističke regresione analize. Prediktivna vrednost biohemijskih indeksa koštanog metabolizma na rizik od preloma OS analizirana je korišćenjem ROC krive.

**Rezultati:**

Grupa sa prelomom OS imala je veći udeo pacijenata sa godinama i nedostatkom sunčeve svetlosti u poređenju sa grupom bez frakture (P < 0,05). Pacijenti u grupi sa frakturom OS su pokazali nižu BMD u lumbalnoj L2č4 i levom femoralnom većem trohanteru u poređenju sa grupom bez preloma (P < 0,05). 14 nedelja i 16 nedelja nakon operacije, nivoi DKK-1, SOST i OPN su bili viši u grupi sa frakturom OS nego u grupi bez frakture, dok su nivoi OPG, BGP i 25 (OH) D bili niži (P < 0,05). BMD u lumbalnom L2'4, BMD u femoralnom velikom trohanteru, OPG, BGP i 25 (OH) D su bili zaštitni faktori (P < 0,05), a starost, nedostatak sunčeve svetlosti, DKK-1, SOST i OPN su bili rizik faktori preloma OS (P < 0,05). BMD u lumbalnom L2'4 je u negativnoj korelaciji sa DKK-1, SOST i OPN (P < 0,05), a u pozitivnoj korelaciji sa BGP i 25 (OH) D (P < 0,05). 25 (OH) D je bio u pozitivnoj korelaciji sa BMD velikog trohantera femura (P < 0,05). OPG, OPN, BGP i 25 (OH) D su imali prediktivnu vrednost za pojavu preloma OS, sa odgovarajućim površinama ispod krive (AUC) od 0,709, 0,761, 0,720 i 0,730. Kada su svi indikatori kombinovani, AUC se povećao na 0,940 (P < 0,05), što označava visoku prediktivnu vrednost za frakture OS.

**Zaključak:**

Indikatori biohemijskog metabolizma kostiju bili su usko povezani sa rizikom od preloma OS i imali su visoku prediktivnu vrednost kao faktori uticaja na pojavu preloma OS. Stoga, tačna kombinacija biohemijskih indeksa može pomoći u smanjenju rizika od preloma kod starijih osoba, omogućavajući razvoj ciljanih planova lečenja za starije pacijente sa prelomima.

## Introduction

Osteoporosis (OS) is a systemic bone disease that is prone to advance to fracture because of the decrease of bone density and bone quality, the destruction of bone microstructure and the increase of bone fragility, which can be caused by various inducements. With the increasing degree of aging, the incidence of OS is also increasing. Moreover, the elderly is prone to fall due to the decline of balance ability, thereby resulting in fracture and increased socio-economic burden.

In recent years, in-depth animal and clinical studies have revealed the pathogenesis of OS fractures, including reduced inflammatory response, decreased mesenchymal stem cells and worsening angiogenesis [1] [2]. Finding approaches to predict the risk of OS fractures in older adults early is important to promote the healing of OS fractures, shorten the length of hospital stay, and reduce related complications. Factors for the development of fractures are complex. Previous studies have found that biochemical indicators of bone metabolism can reflect the activity of osteoblasts and osteoclasts during bone conversion, and the detection of certain biochemical indicators can quickly, sensitively, minimally invasive and reliably predict the risk of fracture in the elderly, so as to effectively evaluate the treatment effect and prognosis of fracture [3]. The measurement of the relationship between SOST and DKK-1 and bone density in bones discover that SOST and DKK-1 in bones are positively correlated with bone density, and higher levels of SOST and DKK-1 may lead to higher bone density, bone microstructure and bone strength [4]. In view of the lack of accurate prediction of a single index, the elderly fracture patients diagnosed and treated in our hospital during 2019 to 2022 were taken as the objects, and the relevant data of patients were collected to analyze the risk factors of OS fracture. We aimed to find a combination of multiple biochemical indicators to improve the sensitivity and accuracy of prediction, thus providing a reliable clinical basis for the prevention and treatment of OS fracture.

## Materials and methods

### General data

**Figure 1:**
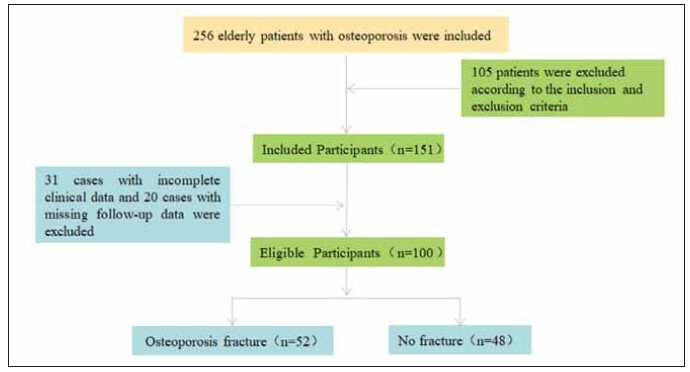
Flow chart of general information selection for 100 patients.

A total of 254 elderly OS patients diagnosed and treated in our hospital during May 2019 to April 2022 was randomly selected, and 100 patients (40 males and 60 females) were finally included in the present study following the inclusion and exclusion criteria. The subjects were aged between 62 to 80 years, with an average age of (72.41 ± 7.82) years. Inclusion criteria: (1) All patients met the diagnostic criteria for OS (5); (2) The patient aged between 60 to 80 years old; (3) All patients have basic literacy and cognitive skills with high degree of cooperation for the research. (4) All patients had not used drugs that could affect outcomes, such as corticosteroids or osteoporosis medications, in the 3 months prior to study participation; (5) All patients and their families agreed to participate in this study and signed an informed consent form. Exclusion criteria: (1) The patients with OS caused by diabetes; (2) The patients with serious obstacles in important organs; (3) The patients who withdrew from the research process; (4) The patients who had the history of tumor and pathologic fractures. The study was approved by the hospital Ethics Committee. Bone density of the patient's lumbar spine was measured, and OS was diagnosed with a 2.5 standard deviation reduction in bone density compared with peak bone mass in healthy young people of the same sex based on the World Health Organization (WHO) standard. According to the presence or absence of OS fractures, patients were randomly divided into OS fracture group and non-fracture group. The osteoporotic fracture group was made up of 25 males and 27 females, with an average age of (83.51 ± 4.28) years. Among them, there were 52 cases in the OS fracture group including 25 males and 27 females, with an average age of (83.51±4.28) years. There were 48 cases in the non-fracture group including 15 males and 33 females, with an average age of (72.41±7.82) years. The process of general data selection was shown in Figure 1.

### Sample collection

The clinical data of patients, including age, gender, height, weight (for the calculation of body mass), smoking history, drinking history, fracture history, lack of sunlight, long-term hormone use, were collected and compared. 5 mL blood was obtained from the patient's elbow vein and centrifuged at room temperature at 3000 r/min to isolate the serum. The separated serum was stored at -80°C for following detections. The blood of elderly fracture patients was collected on the day of admission, the first day, first week, second week, fourth week, twelfth week and sixteenth week after operation. The blood of elderly without fracture was collected only once.

### Outcome measures

(1) The bone mineral density (BMD) of lumbar L2~4 and left femoral greater trochanter of the patients was measured using the dual energy X-ray BMD instrument (Lunar DPXIQ No.5689, USA).

(2) The levels of bone metabolism biochemical indexes, including Dickkopf-1 (DKK-1), sclerostin (SOST), osteoprotegerin (OPG), osteopontin (OPN), osteocalcin (BGP) and 25 hydroxyvitamin D (25 (OH) D) were detected by enzyme-linked immunosorbent assay. Each indicator was measured three times repeatedly and averaged.

### Statistical analysis

Statistic Package for Social Science (SPSS) 23.0 data statistics software (IBM, Armonk, NY, USA) was used for the data statistics. The enumeration data, such as gender, smoking history, drinking history and fracture history were expressed in (cases (%)) and compared using 2 test. Measurement data, such as BMD and bone biochemical indicators were tested by normal distribution, and they all conformed to normal distribution. The measurement data were expressed by (x̄±s) and compared using t test between two groups or ANOVA among multiple groups. The influencing factors of OS fracture were analyzed using Multivariate logistic regression analysis. The relationship between biochemical indexes of bone metabolism and BMD was analyzed using Pearson correlation analysis. The clinical value of biochemical indicators of bone metabolism in predicting the occurrence of OS was analyzed using ROC curve. P < 0.05 indicated that the difference was statistically significant.

## Results

Clinical data, medical history and changes of BMD in two groups

The proportion of patients with age and lack of sunlight in the OS fracture group was significantly higher than that in the non-fracture group (P < 0.05, Table 1). The BMD in lumbar L2~4 and left femoral greater trochanter of patients in the OS fracture group was prominently lower than that of patients in the non-fracture group (P < 0.05, Table 1).

**Table 1:** Clinical data, medical history and changes of BMD in two groups (x̄±s).

Items		OS fracture group <br>(n=52)	Non-fracture group <br>(n=48)	χ^2^/t	p
Age (year)		83.51±4.28	72.41±7.82	8.896	<0.001
Gender	Male	25 (48.08)	15 (31.25)	2.945	0.086
	Female	27 (51.92)	33 (68.75)		
Weight (kg)		56.70±8.25	57.88±10.12	0.641	0.523
BMI (kg/m^2^)		22.30±3.42	22.89±5.46	0.653	0.515
Smoking history	Yes	20 (38.46)	21 (43.75)	0.289	0.591
	No	32 (61.54)	27 (56.25)		
Drinking history	Yes	8 (15.38)	10 (20.83)	0.502	0.479
	No	44 (84.62)	38 (79.17)		
Lack of sunshine <br>exposure	Yes	30 (57.69)	11 (22.92)	12.478	<0.001
	No	22 (42.31)	37 (77.08)		
History of fracture	Yes	6 (11.54)	8 (16.67)	0.545	0.460
	No	46 (88.46)	40 (83.33)		
Long term hormone <br>administration	Yes	1 (1.92)	0 (0.00)	0.951	0.330
	No	51 (98.08)	48 (100.00)		
BMD of Lumbar L2~4 (g/cm^2^)		0.90±0.14	1.24±0.15	11.724	<0.001
BMD of greater trochanter of femur (g/cm^2^)		0.68±0.11	0.76±0.12	3.478	0.001

### Changes of biochemical indexes of bone metabolism

Compared with these at admission, the levels of DKK-1, SOST and OPN in the two groups were observably increased on the first day after operation, while these of OPG, BGP and 25 (OH) D sharply decreased (P < 0.05). With the extension of postoperative time, the levels of dickkopf-1, SOST and OPN gradually decreased, whereas these of OPG, BGP and 25(OH) D gradually increased (P < 0.05). At 14 and 16 weeks after operation, the levels of DKK-1, SOST and OPN of patients in the OS fracture group was higher than these of patients in the non-fracture group, while inverse results were observed in the levels of OPG, BGP and 25 (OH) D in two groups (P < 0.05, Table 2).

**Table 2:** Changes of biochemical indexes of bone metabolism (x̄±s) Note: aP < 0.05 compared with non-fracture group.

Groups	Cases	Time	DKK-1 <br>(ng/mL)	SOST <br>(ng/mL)	OPG (μg/L)	OPN (ng/mL)	BGP (μg/L)	25 (OH) D <br>(ng/mL)
OS fracture <br>group	52	On admission	3.25±1.20^a^	1.32±0.65^a^	37.39±10.41^a^	11.02±2.45^a^	37.04±5.42^a^	10.35±3.57^a^
		1 day after <br>operation	3.57±1.02	1.59±0.74	34.32±10.18	14.52±3.50	34.72±4.52	8.49±2.30
		1 week after <br>operation	3.41±1.03	1.44±0.56	36.83±12.29	13.74±3.22	35.90±5.10	9.43±3.98
		2 weeks after <br>operation	3.25±1.20	1.31±0.62	40.46±14.17	12.85±3.68	37.23±5.14	10.58±4.52
		4 weeks after <br>operation	3.16±1.02	1.23±0.46	43.95±14.82	12.01±2.73	39.02±7.95	11.64±4.02
		12 weeks after <br>operation	3.13±1.09^a^	1.12±0.41^a^	44.45±17.75^a^	12.23±3.07^a^	41.07±6.97^a^	13.11±6.57^a^
		16 weeks after <br>operation	3.12±1.25^a^	1.10±0.54^a^	47.23±16.97^a^	12.44±3.18^a^	41.19±8.23^a^	13.83±6.33^a^
Non-fracture <br>group	48	On admission	3.33±1.89	1.31±0.58	36.42±8.56	11.39±1.87	38.26±6.57	11.02±3.48
		1 day after <br>operation	3.60±1.18	1.58±0.62	33.25±10.20	13.52±3.44	35.61±4.89	8.52±2.49
		1 week after <br>operation	3.39±1.21	1.43±0.60	36.02±11.32	13.02±2.48	36.23±6.42	9.33±3.82
		2 weeks after <br>operation	3.24±1.18	1.30±0.52	41.25±9.85	12.21±4.26	37.44±5.26	10.60±4.32
		4 weeks after <br>operation	3.13±1.05	1.22±0.45	44.33±12.25	11.58±3.06	40.63±4.85	13.26±4.67
		12 weeks after <br>operation	2.61±1.15	0.93±0.42	52.20±10.69	10.12±3.69	43.26±7.59	15.88±5.26
		16 weeks after <br>operation	2.55±1.30	0.88±0.31	55.75±14.83	9.39±4.41	45.28±7.14	17.19±4.07

### The risk factors for the occurrence of OS fractures by the Multivariate Logistic regression analysis

Logistic regression analysis showed that BMD in lumbar L2~4, BMD in femoral greater trochanter, OPG, BGP and 25 (OH) D were the protective factors (P < 0.05), and the age, lack of sunlight, DKK-1, SOST and OPN were the risk factors affecting OS fractures in the elderly (P < 0.05, Table 3).

**Table 3:** Multivariate Logistic regression analysis on risk factors of OS fracture.

Indicators	B value	SE	Wald value	P value	OR value	95% CI	
						Lower limit	Upper limit
Age	1.105	0.638	2.715	0.047	1.014	1.008	4.218
Lack of sunlight	2.602	0.678	5.657	0.001	13.492	3.671	19.258
BMD of Lumbar L2~4	-2.339	0.925	2.732	0.028	0.784	0.117	0.851
BMD of greater <br>trochanter of femur	-1.442	0.705	2.901	0.024	0.237	0.062	0.935
DKK-1	1.158	0.554	3.772	0.004	1.557	1.477	12.267
SOST	3.832	0.832	5.538	0.007	8.176	2.719	10.058
OPG	-0.672	0.342	5.744	0.001	0.521	0.392	0.672
OPN	2.207	0.782	3.606	0.007	7.230	1.798	13.851
BGP	-2.004	0.472	8.987	0.001	0.135	0.053	0.340
25 (OH) D	-0.587	0.233	10.870	0.001	0.625	0.079	0.895

### Correlation between biochemical indexes of bone metabolism and BMD in OS fractures

**Figure 2:**
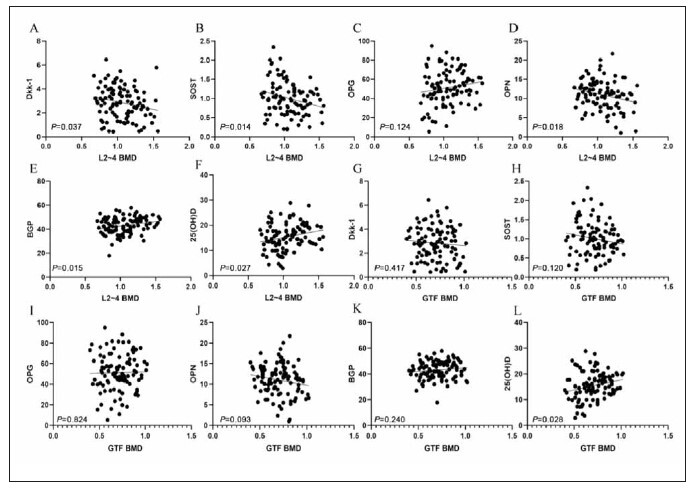
Correlation between biochemical indexes of bone metabolism and BMD in OS fracture. (A~F) Correlation between biochemical indexes of bone metabolism and lumbar L2~4 BMD in OS fracture. (G~L) Correlation between biochemical indicators of bone metabolism and BMD of femoral trochanter in OS fracture.

Pearson correlation analysis revealed that BMD of Lumbar L2~4 BMD was negatively correlated with DKK-1, SOST and OPN (P < 0.05), and positively correlated with BGP and 25 (OH) D (P < 0.05). 25 (OH) D was positively correlated with BMD of femoral greater trochanter (P < 0.05, Table 4 and Figure 2).

**Table 4:** Correlation between biochemical indexes of bone metabolism and BMD in OS fracture.

Items	BMD of Lumbar L2~4 BMD		BMD of femoral greater trochanter	
	r	P	r	P
DKK-1	-0.209	0.037	-0.082	0.417
SOST	-0.246	0.014	-0.157	0.120
OPG	0.155	0.124	0.023	0.824
OPN	-0.236	0.018	-0.169	0.093
BGP	0.243	0.015	0.119	0.240
25 (OH) D	0.221	0.027	0.221	0.028

### Analysis of the predictive value of biochemical indicators of bone metabolism on the risk of OS fracture

**Figure 3:**
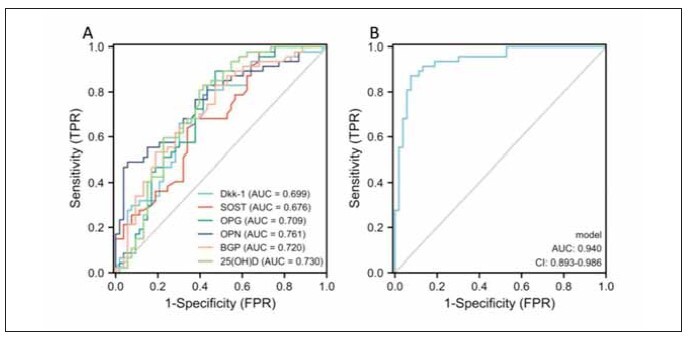
Analysis of the predictive value of biochemical indicators of bone metabolism on the risk of OS fracture using ROC curve. (A) Single index detection; (B) Combined detection of all indicators. Analysis of the predictive value of biochemical indicators of bone metabolism on the risk of OS fracture using ROC curve. (A) Single index detection; (B) Combined detection of all indicators.

ROC curve analysis showed that OPG, OPN, BGP and 25 (OH) D had certain predictive value for the occurrence of OS fracture in the elderly with the areas under the curve (AUC) of 0.709, 0.761, 0.720 and 0.730, respectively. The combined detection of all indicators had the AUC of 0.940 (P < 0.05, Table 5 and Figure 3), which had a high predictive value for OS fracture.

**Table 5:** Analysis of the predictive value of biochemical indicators of bone metabolism on the risk of OS fracture.

Indicators	AUC	95%CI		Sensitivity	Specificity	P
		Lower limit	Upper limit			
DKK-1	0.699	0.596	0.803	80.9	56.6	0.034
SOST	0.676	0.571	0.780	68.1	62.3	0.007
OPG	0.709	0.606	0.811	89.4	52.8	0.014
OPN	0.761	0.666	0.856	48.9	94.3	0.003
BGP	0.720	0.620	0.821	83.0	52.8	0.001
25 (OH) D	0.730	0.629	0.830	83.0	58.5	0.011
Combined	0.940	0.893	0.986	87.2	92.5	<0.001

## Discussion

Osteoporosis (OS) fracture, also known as fragile fracture, is easy to occur in the elderly. Forearm, hip and vertebra are the most vulnerable parts to fracture. At the same time, due to the poor immune function and the frequent combination of many chronic diseases, the elderly patients with OS fracture are often accompanied by high disability rate and fatality rate, which affects the life of patients [5] [6].

BMD is an established determinant of bone strength. An individual's bone density in later life depends on the peak bone growth achieved at age 40 and the subsequent rate of bone loss. Decreased BMD is an important determinant of OS fractures, and the measurement of BMD is a key component in diagnosing OS [7]. The previous study has proved that bone density and a history of non-vertebral fractures can predict the fracture of a large number of postmenopausal women for up to 20~25 years [8]. In addition, low bone density is associated with a higher risk of fracture in people with diabetes compared with non-diabetics [9]. BMD can also be used for follow-up of anti-OS fracture treatment in patients with diabetes. In this study, the BMD level of lumbar L2~4 and left femoral greater trochanter in elderly patients with or without fracture was analyzed. The results showed that the BMD of L2~4 of lumbar vertebrae and the greater trochanter of left femur in patients with fracture strongly decreased. Thus, the BMD of lumbar L2~4 and left femoral greater trochanter may be important auxiliary indexes to evaluate the fracture.

Bone metabolism is the process of bone resorption and bone formation, and the biochemical indexes of bone metabolism play an important role in the activity of bone cells in response to bone conversion. SOST, DKK-1, OPG, OPN, BGP and 25 (OH) D are all bone biochemical metabolic indexes commonly used in clinical practice to assess fracture risk, which play a crucial role in maintaining bone homeostasis. The results from the present study showed that BMD in lumbar L2~4, BMD in femoral greater trochanter, OPG, BGP and 25 (OH) D were the protective factors, and the age, lack of sunlight, DKK-1, SOST and OPN were the risk factors affecting OS fractures in the elderly. Moreover, the biochemical indexes of bone metabolism, such as OPN and BGP were significantly correlated with BMD. SOST is a secreted glycoprotein that is widely found in the lungs, kidneys and bones. SOST may be involved in pathophysiological processes such as fracture healing and disc degeneration, which may be achieved through its angiogenesis [10]. DKK-1 is a recognized classical Wnt signaling inhibitor that is fully participates in the regulation of bone formation and is implicated with the occurrence and progression of bone metastases. In patients with diabetes, DKK-1 levels are significantly elevated, which is significantly associated with decreased BMD [11]. In bone tissue, bone resorption by osteoclasts and bone formation by osteoblasts are constantly repeated. Osteoclasts are multinucleated cells that originate from monocytes/macrophage lineage cells and absorb bone. In contrast, osteoblasts mediate osteoclastigenesis by expressing receptor activators of nuclear factor- B ligand (RANKL), which is expressed as membrane-associated cytokines [12]. OPG is a soluble RANKL bait receptor produced primarily by osteoblasts that prevents osteoclast formation and osteoclast bone resorption by inhibiting RANKL-RANKL receptor interactions. It has been revealed that the OPG levels are significantly negatively correlated with the incidence of OS fractures in older adults [13]. BGP is a special bone protein synthesized by odentinocytes and osteoblasts, mainly secreting noncollagen, and is currently considered a specific indicator of the rate of bone formation [14]. The study has found that serum BGP can effectively predict the risk of fracture in patients with OS, which is of great value for the prevention of OS fractures in the elderly [15].

As a transformation-related phosphoprotein, OPN has been proven to be closely related to the occurrence and development of various bone-related diseases such as osteoporosis, rheumatoid arthritis, and osteosarcoma [16]. Vitamin D is a precursor to 25 (OH) D and other metabolites, and the effects of the vitamin D endocrine system on bones and their growth plates are primarily indirect and mediated by their effects on intestinal calcium transport and serum calcium and phosphate homeostasis. Studies have shown that 25 (OH) D affects local bone metabolism and BMD microstructure, thus increasing the risk of OS fractures [17]. Timely detection and intervention according to the above risk factors can avoid or reduce the risk of osteoporotic fractures as much as possible. In addition, the ROC curve confirmed that a variety of biochemical indexes of bone metabolism such as SOST and DKK-1 could predict the risk of OS fractures in the elderly, and the combined detection value was higher, indicating that the level of biochemical indexes of bone metabolism played a significant role in predicting the risk of OS fractures in the elderly.

In summary, the risk of OS fracture in the elderly has a significant correlation with biochemical indexes of bone metabolism, which is an affecting factor for the risk of OS fracture in the elderly. Moreover, the combined detection of biochemical indexes of bone metabolism has a high predictive value in the risk of OS fracture in the elderly. This study can provide clinical guidance strategies for the prevention of fractures in elderly fracture patients. However, a more accurate threshold could not be proposed due to the small sample size in the current study. As a detection method and research method, joint prediction provides the right direction for future research. Additionally, the relatively short follow-up duration may also lead to the potential missing of patients who could experience adverse outcomes. However, the ultimate predictor of the risk of OS fractures in older adults requires a large, multicenter randomized controlled study to reach a clinical consensus.

## Dodatak

### Funding

This work was supported by Hunan Provincial and Municipal Natural Science Foundation (No.2021JJ50064).

### Availability of data and materials

The datasets used and/or analyzed during the current study are available from the corresponding author on reasonable request.

### Conflict of interest statement

All the authors declare that they have no conflict of interest in this work.
